# Streptococcus pyogenes M1UK Variant-Associated Sartorius Muscle Necrotizing Soft Tissue Infection: A Case Report and Literature Review

**DOI:** 10.7759/cureus.75765

**Published:** 2024-12-15

**Authors:** Kyoko Baba, Risako Ito, Yuki Ando, Haruno Yoshida, Takashi Takahashi

**Affiliations:** 1 Department of Plastic and Aesthetic Surgery, Kitasato University School of Medicine, Sagamihara Kanagawa, JPN; 2 Department of Plastic Surgery, Kitasato University Medical Center, Kitamoto, JPN; 3 Laboratory of Infectious Diseases, Graduate School of Infection Control Sciences & Ōmura Satoshi Memorial Institute, Kitasato University, Tokyo, JPN

**Keywords:** m1uk-lineage, necrotizing soft tissue infection, sartorius muscle, skin graft, streptococcus pyogenes

## Abstract

Necrotizing soft tissue infections (NTSIs) represent a concept of necrotizing infections involving the skin, subcutaneous tissue, fascia, and muscle, and it is a potentially fatal disease. Early exploratory incision is strongly recommended for both the diagnosis and treatment of necrotizing soft tissue infections. Treatment of necrotizing soft tissue infections requires the administration of appropriate antimicrobial agents and adequate surgical debridement. The emergence of M1_UK_-lineage *Streptococcus pyogenes* (*S. pyogenes*) is recently reported in the UK, Canada, the USA, and the Netherlands. We report a Japanese case of sartorius muscle (SM) NTSI caused by M1_UK_-lineage *S*.* pyogenes*. A 34-year-old man developed redness and swelling of his right thigh anterior compartment with fever in October 2024. The closed and deep effusions by active exploratory incision/debridement on hospital days one to three yielded the presence of Gram-positive cocci, although two sets of blood cultures upon admission revealed no bacterial growth; its species identification results indicated *S*. *pyogenes*. Clinical and pathological diagnosis was streptococcal SM NTSI (without toxic shock syndrome (TSS)). Negative pressure wound therapy with instillation and dwelling (NPWTi-d) to promote his soft tissue cure was performed along with antimicrobial regimens. The patient recovered and received micrografting (carrier: artificial dermis made from collagen sponge and silicon film, Pelnac Gplus® (Gunze Limited, Osaka, Japan) using the Rigenera® system (Rignera HBW, Candiolo, Italy). Thereafter, he developed bronchitis: the sputum yielded *S*. *pyogenes* growth: he recovered uneventfully. Split-thickness skin grafting (STG) was performed. Split-thickness skin grafting was fully engrafted, and the wounds achieved complete healing. The patient could walk by himself. Microbiological genetic analyses using both DNAs from effusion/sputum-origin strains revealed the *emm1.0* and *speA*-*speB*-*smeZ* profiles. *rofA*-*gldA*-*pstB* sequencing results indicated M1_UK_-specific single-nucleotide polymorphisms. The streptococcal inhibitor of the complement-mediated lysis gene allele was the streptococcal inhibitor of the complement-mediated lysis-1.02 allele. Micrografting using the Rigenera® system and STG following NPWTi-d can be beneficial approaches. Clinicians should perform cultures using sterile specimens (deep effusions/tissues) from infection foci through exploratory incision/debridement, along with two sets of blood cultures, when examining patients with/without underlying medical conditions.

## Introduction

Necrotizing soft tissue infections (NSTIs) represent a concept of necrotizing infections involving the skin, subcutaneous tissue, fascia, and muscle [1]. A NSTI is a potentially fatal disease. Early exploratory incision is strongly recommended for both the diagnosis and treatment of NSTI [1]. Key diagnostic findings during exploratory incision include the presence of "dishwater" fluid, a positive "finger test," scant soft tissue hemorrhage, and weak muscle contraction.

As a supplementary diagnostic tool, the Laboratory Risk Indicator for Necrotizing Fasciitis (LRINEC) score is relatively well recognized. Developed by Wong et al. [2], the LRINEC score is designed to distinguish NSTI from other soft tissue infections. It incorporates six laboratory parameters: white blood cell (WBC) count, hemoglobin, sodium, glucose (≦180 or >180 mg/dL), creatinine (≦1.6 or >1.6 mg/dL), and C-reactive protein. The maximum score is 13, with a score of ≧6 indicating a 50%-75% probability of NSTI and a score of ≧8 being highly predictive of NSTI, with a probability exceeding 75%. However, the LRINEC score may have limitations in accurately differentiating NSTI. Histopathological findings specific to NSTI are generally absent, though prominent features such as strong inflammatory cell infiltration and destruction of fascia and muscle are often observed. In addition to the critical findings obtained through exploratory incision, NSTIs are diagnosed clinically through a comprehensive evaluation of the LRINEC score, clinical progression, imaging findings, laboratory results, bacterial cultures, and pathological findings. Treatment of NSTI requires the administration of appropriate antimicrobial agents and adequate surgical debridement.

The sartorius muscle (SM) with the quadriceps femoris constitutes the thigh anterior compartment of human beings. As an anatomical feature, SM is the longest muscle of the human body, in which SM originates from the anterior superior iliac spine and terminates into the medial side of the tibia. An NSTI in the SM is rare.

*Streptococcus pyogenes* (*S. pyogenes*) exhibiting Lancefield carbohydrate A antigen with beta-hemolysis is one of the major Gram-positive cocci in human clinical settings. Skin/soft tissue illnesses (including erysipelas, contagious impetigo, and NSTI) are the most common disease entities, whereas primary peritonitis or empyema are rare conditions. Unfortunately, some patients may develop streptococcal toxic shock syndrome (STSS), leading to shock and multi-organ failure. Recently, the emergence of M1_UK_-lineage *S. pyogenes* has been reported in the UK, Canada, the USA, and the Netherlands [3-6]. This M1T1 variant can possess toxigenic and hypervirulent properties, which include the enhanced production and secretion of streptococcal pyrogenic exotoxin A (also known as scarlet fever or erythrogenic toxin A), and can develop the increased incidence of scarlet fever among children. This variant also has several genes encoding virulence factors involved in the adherence, immune evasion, and pathogenicity that the contemporary strains (M1_global_-lineage) possess.

We, herein, describe a case of SM NSTI caused by M1_UK_-lineage *S. pyogenes* in a man in Japan. Furthermore, the literature review, including main case reports of the M1_UK_-associated infections, is represented in the discussion section.

## Case presentation

**Figure 1:**
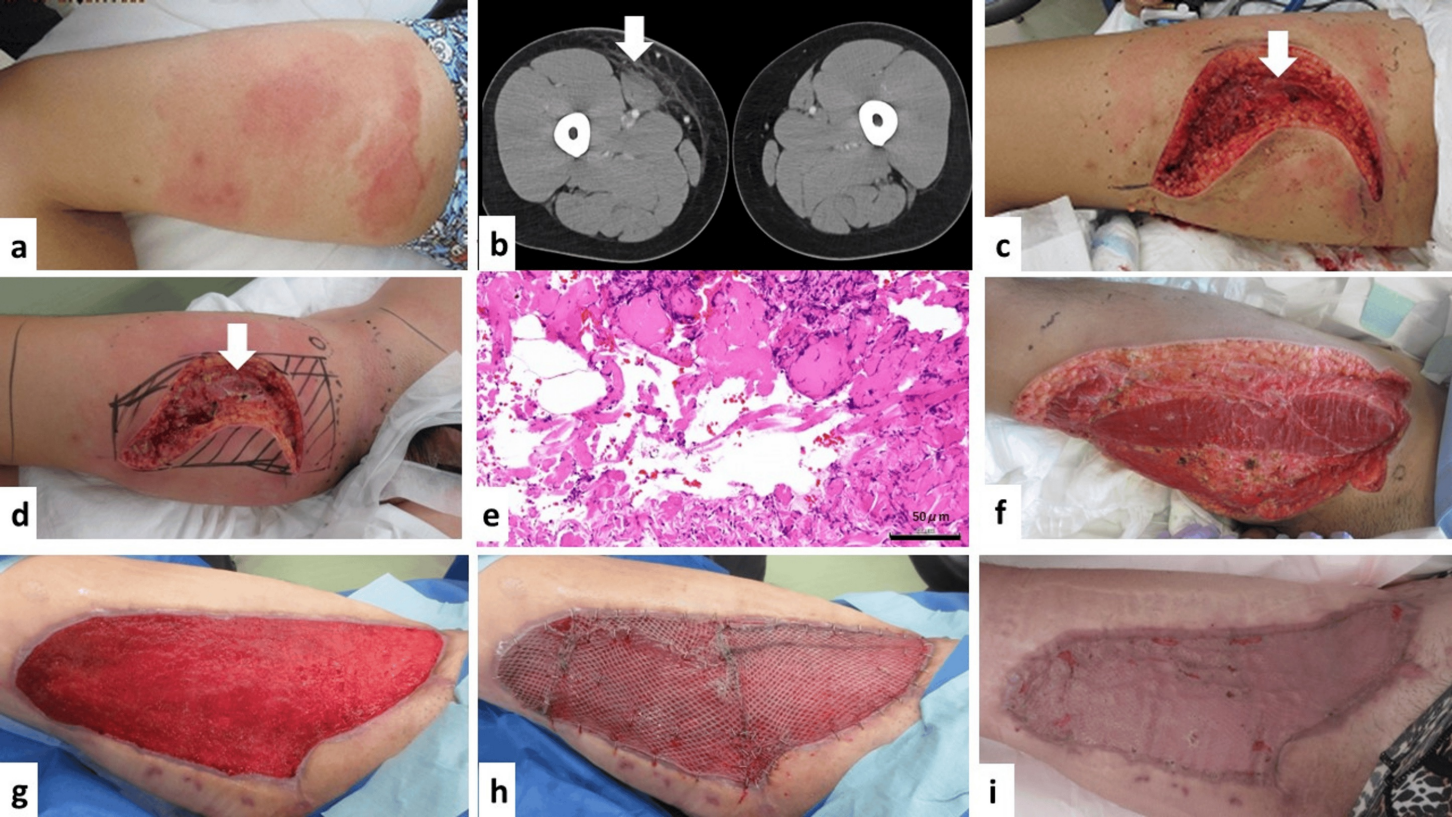
Clinical course of necrotizing soft tissue infection (NSTI) at right sartorius muscle (SM) (a) Physical examination upon admission was notable for redness, swelling, and tenderness of his right thigh anterior compartment. (b) Computed tomography images of the patient’s bilateral lower limbs upon admission revealed the partially attenuated low density (arrow) at the right SM, suggesting its compatibility with NSTI. (c) Active exploratory incision lines and appearances were noted at the right thigh area upon admission. The arrow reveals the SM fascia. (d) Debridement was conducted on hospital day two. Arrow reveals the SM fascia. (e) Pathological findings were obtained through the debridement procedure, which was compatible with necrotizing soft tissue infection (bar: 50 μm). There was muscular fiber damage with cell debris. (f) Wound conditions with good local management are noted on hospital day seven. Almost all anterior compartments of the SM fascia were resected with partial compartments of rectus femoris muscle/vastus medialis muscle fascia. (g) Wound conditions before completing split-thickness skin grafting (STG) on hospital day 39. Good granulation covered the wound lesion. There was no difference in level between the wound and surrounding normal skin surfaces. (h) The STG was conducted on hospital day 39. The patient's skin graft was composed of thickness (12/1000 inch), area (approximately 300 cm²), and mesh (1.5-fold). (i) The STGs were fully engrafted, and the wounds achieved complete healing.

A 34-year-old man developed pain, redness, and swelling in his right thigh area with diarrhea, sore throat, cough, and fever in October 2024. He was treated in the Department of Plastic Surgery at Kitasato University Medical Center. His medical history included atopic dermatitis. His child had already suffered from contagious impetigo and had received its treatment at a pediatric clinic. All rapid antigen tests to detect group A Streptococcus, SARS-CoV-2, and influenza virus using the pharyngeal swab, nasal swab, and nasopharyngeal swab were negative. His vital signs (consciousness, respiratory rate, oxygen saturation at room air, and heart rate upon admission) were stable except for a high temperature (40.5 °C) and hypotension. Physical examination upon admission was notable for redness, swelling, and tenderness of his right thigh anterior compartment (Figure 1a). Laboratory blood tests upon admission showed an elevated WBC count of 13,340/μL (normal range, 4,000-9,000/μL) and an elevated serum C-reactive protein level at 20.64 mg/dL (normal range, 0-0.3 mg/dL) with a slightly increased serum creatine kinase value of 830 U/L (normal range, 60-247 U/L): the LRINE score reached six. Computed tomography (CT) images of his bilateral lower limbs upon admission (Figure 1b) revealed the partially attenuated low density at the right SM, suggesting that it was compatible with NSTI. Active exploratory incision lines and appearances upon admission (Figure 1c) indicated the vulnerable connective tissue strength (a positive finger test) along with the effusion around the fascia.

As initial antimicrobials, meropenem (at a dosing of 3 g/day) along with clindamycin (at a dosing of 2.7 g/day) were intravenously administered to him. We carried out the subsequent twin debridement on hospital days two (Figure 1d) and three for active treatment of the infection focus.

Although two sets of blood cultures upon admission revealed no bacterial growth, the closed and deep effusions obtained through active exploratory incision and subsequent twice debridement on hospital days one, two, and three yielded the presence of Gram-positive cocci on their staining. Its subsequent species identification and antimicrobial susceptibility pattern based on the broth microdilution method (according to Clinical and Laboratory Standards Institute document M100-S25) were performed using MicroScan WalkAway (Beckman Coulter, Inc., Tokyo, Japan). These results indicated *S. pyogenes* having the pattern susceptible to all validated antimicrobials (one strain designated as JN1-1), although the minimum inhibitory concentration of minocycline against this strain revealed 2 μg/mL. His clinical diagnosis was considered NSTI of SM without STSS caused by *S. pyogenes*. Accordingly, the antimicrobial regimen was changed to cefotaxime (at a dosing of 4 g/day) alone. We confirmed that the pathological findings obtained through the debridement procedure (Figure 1e) were compatible with NSTI.

Figure 1f indicates the wound conditions with good local management on hospital day seven. Almost all of the anterior compartments of the SM fascia were resected along with partial compartments of the rectus femoris muscle/vastus medialis muscle fascia. On hospital day eight, negative pressure wound therapy with instillation and dwelling time (NPWTi-d; 3M™ V.A.C.® Ulta Therapy System, Solventum, St Paul., MN, USA) to promote his soft tissue cure was initiated and continued. The patient recovered and received the micrograft treatment with the Rigenera® system (Rignera HBW, Candiolo, Italy) using an artificial dermis made of collagen sponge and silicone film (Pelnac Gplus®, Gunze Limited, Osaka, Japan) as a carrier and scaffold on hospital day 16. We found the growth of granulation at the wound through NPWTi-d, and the granulation covered the SM lesion on hospital day 25.

On hospital day 29, the patient had a productive cough and developed bronchitis without abnormal chest roentgenogram findings. The sputum culture indicated *S. pyogenes* having the same susceptible pattern (another strain designated as JN1-2). He was orally administered clindamycin (at a dosing of 0.9 g/day) and recovered uneventfully.

To perform the wound closure on hospital day 39, split-thickness skin grafting (STG) was carried out after conducting the wound debridement (Figures 1g-1h). His skin graft was obtained from the left thigh anterior and lateral compartments, which were composed of the thickness (12/1000 inch), area (approximately 300 cm2), and netlike tissue (1.5-fold). The NPWTi-d was restarted after completing STG immediately. The post-STG course was uneventful; the STG was fully engrafted, and the wounds achieved complete healing (Figure 1i). The patient could walk by himself during discharge.

Microbiological genetic analyses

The microbiological genetic analyses were conducted at the Laboratory of Infectious Diseases, Graduate School of Infection Control Sciences, and the Ōmura Satoshi Memorial Institute, Kitasato University. We included American Type Culture Collection 12344(T) as a positive control of *S. pyogenes*. The DNAs were extracted from the strains by suspending them in Tris-EDTA buffer and boiling them at 97°C for 10 minutes [7]. We determined the genotype of *emm* (encoding surface filamentous M protein) and the profile of the exotoxin gene (consisting of *speA*-*speB*-*speC*-*ssa*-*smeZ*) as previously described [8]. According to the published articles [9,10], allele-specific polymerase chain reaction (PCR) with direct sequencing of *rofA* (encoding a positive regulator of pilus locus)-*gldA* (encoding glycerol dehydrogenase)-*pstB* (encoding phosphate ABC transporter ATP-binding protein) was conducted to identify the M1_UK_-specific single-nucleotide polymorphisms (SNPs). We also determined the streptococcal inhibitor of complement-mediated lysis (*sic*) gene allele [8] and its antimicrobial resistance (AMR) gene profile [*blaZ*-*erm*(A)-*erm*(B)-*mef*(A)-*linB*-*lnuD*-*tet*(M)-*tet*(O)-*tet*(K)-*tet*(L)-*tet*(S)] [11]. Sequencing of the PCR-positive products was carried out on Applied Biosystems 3730xl DNA Analyzer with BigDye Terminator V3.1 (Thermo Fisher Scientific, Waltham, MA, USA).

**Figure 2:**
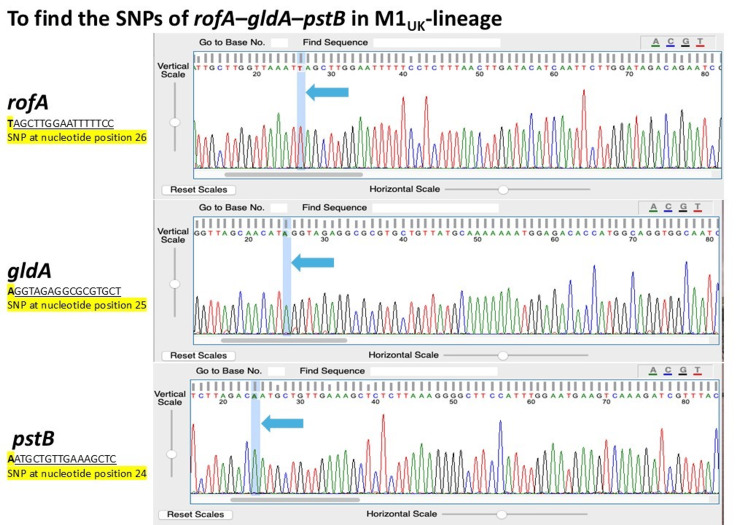
M1UK-specific single-nucleotide polymorphisms (SNPs) of rofA (encoding a positive regulator of pilus locus)–gldA (encoding glycerol dehydrogenase)–pstB (encoding phosphate ABC transporter ATP-binding protein) on ABI sequence chromatograms. Blue highlights (arrows) show the three SNPs of upper rofA, middle gldA, and lower pstB.

**Figure 3:**
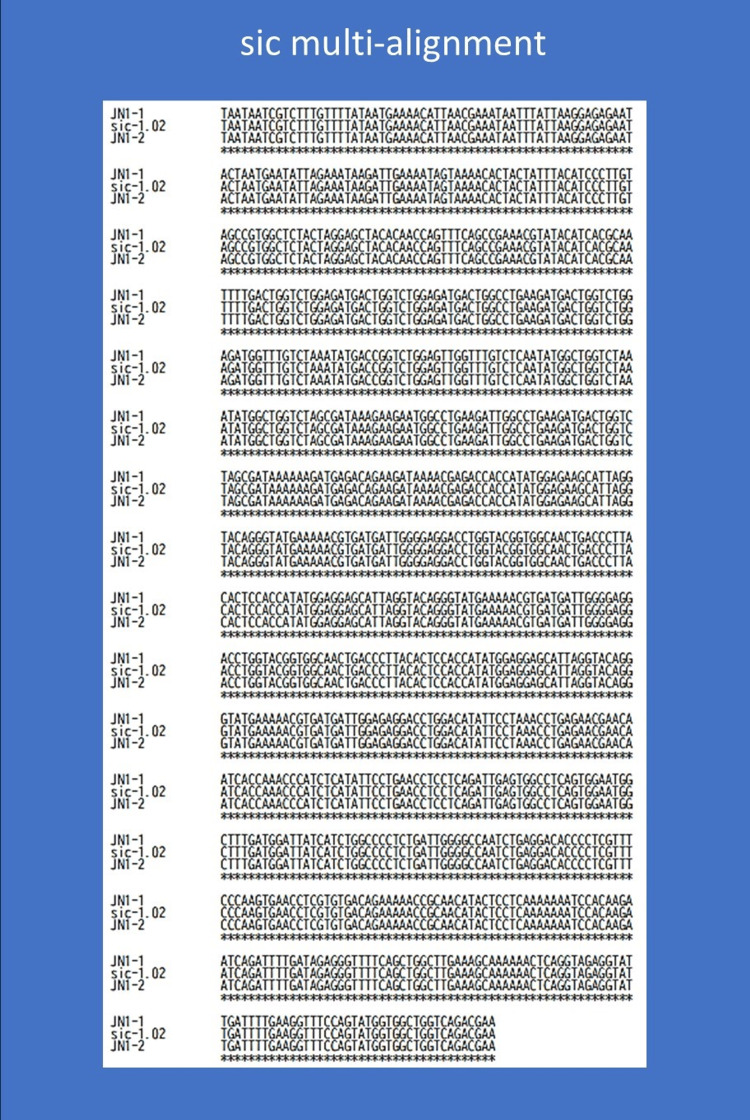
Multi-alignment of streptococcal inhibitor of complement-mediated lysis gene from both JN1-1 and JN1-2 generated using Multiple Sequence Alignment by CLUSTALW. The corresponding streptococcal inhibitor of complement-mediated lysis (sic) gene allele number was the identical sic-1.02 allele (sequencing size 940 bp); https://www.genome.jp/tools-bin/clustalw [12]

Genetic analyses using both DNA specimens from JN1-1 and JN1-2 revealed the *emm*1.0 and *speA*-*speB*-*smeZ* profiles (without *speC*-*ssa* detection). The *rofA*-*gldA*-*pstB* sequencing results from JN1-1 and JN1-2 indicated the allele-specific SNPs (Figure 2 showing the SNPs from JN1-1). The sic allele number from both JN1-1 and JN1-2 was found to be the identical *sic*-1.02 allele (sequencing size 940 bp) (shown in Figure 3), and we did not detect AMR genes.

## Discussion

To diagnose NSTI, it is crucial to maintain a high index of suspicion for this life-threatening condition at an early stage. While certain risk factors and predispositions, such as advanced age, diabetes, immunosuppression, cancer, and liver cirrhosis, are well-documented, it is important to recognize that NSTI can also develop in young, healthy individuals without any apparent trauma or predisposing factors [1]. Local clinical findings of NSTI include intense pain disproportionate to visible skin findings, edema, tenderness, and induration (including lymphadenopathy) beyond the area of visible erythema. Necrotizing soft tissue infections typically progress through three stages: stage 1 (early), characterized by tenderness, erythema, swelling, and warmth; stage 2 (intermediate), marked by the appearance of bullae, skin fluctuation, and induration; and stage 3 (late), featuring serous bullae, diminished skin sensation, crepitus, and necrosis [1]. Although early differentiation of NSTI from other soft tissue infections can be challenging, prompt exploratory incision significantly aids in diagnosis and improves patient outcomes [1]. Thus, this surgical approach should not be delayed. The progression of NSTI can vary, ranging from rapid to relatively gradual, and a transition from slow to sudden progression is also possible. Patients presenting with severe systemic symptoms, such as fever or altered mental status, are more likely to have NSTI. For such cases, we actively perform exploratory incisions to achieve timely diagnosis, which is critical for limb preservation and survival. In our case, NSTI primarily involved the SM fascia in an adult patient without any known risk factors or predisposing conditions. Moreover, the site of infection was highly unusual. However, an internist who initially examined the patient promptly included NSTI in the differential diagnosis and immediately referred the patient to our department. This early identification and timely surgical intervention were key to the favorable outcome.

At our institution, we have observed a growing number of NSTI cases in recent years, with 37% of patients initially presenting to internal medicine departments. This underscores the importance of NSTI awareness across all specialties.

Effective treatment of NSTI requires both appropriate antibiotic therapy and debridement of necrotic tissue. Reconstruction of tissue defects is also necessary, but wound closure should ideally be considered only after infection control has been achieved. Inadequate debridement, followed by premature wound closure, risks persistent infection and worsening of the condition. In our case, even after infection control appeared successful, close monitoring of the wound was maintained. The NPWTi-d facilitates regular irrigation of the wound, while micrografting promotes wound healing by inducing cytokines and other factors without complete wound closure [13]. We combined these advanced wound management strategies, which expedited healing. Additionally, the use of artificial dermis for wound repair achieved functional and aesthetic results acceptable to the patient, avoiding tissue contracture or depression. Consistent monitoring and infection control remain indispensable. However, as with our prior research [13], the combination of NPWTi-d and micrografting proved effective in the present case.

The most well-known causative pathogen of NSTI is Group A Streptococcus; however, a variety of other pathogens, including anaerobes and enterobacteria, have been reported, and polymicrobial infections are not uncommon. In our case, the causative organism was identified as *S. pyogenes* of the M1_UK_ lineage. To the best of our knowledge, no prior reports of NSTI caused by this specific strain have been identified in the literature.

Table 1 summarizes the literature review, including the main four case reports of the M1_UK_-associated infections among adults [10,14]. This table contains the publication year, country, sex, age, comorbidities, isolation source(s), clinical diagnosis, initial antimicrobial(s), antimicrobial susceptibility, surgical intervention(s), other intervention(s), and outcome. Out of four patients, there were two cases of pneumonia due to the M1_UK_-lineage. The relationships between pulmonary infections and M1_UK_-lineage are reported in the UK [15], Portugal [16], and Belgium [17]. In central Scotland, there was an increase in severe pulmonary infections among adults due to the M1_UK_ variant, which were associated with influenza A co-infection and fatality rates [15]. There was also an increase in pediatric invasive *S. pyogenes* infections dominated by the M1_UK_ variant in Portugal (n = 89) [16]. From September 2022 through May 2023, the dominant diagnosis was pneumonia (25/79), mostly with empyema (20/25). A number of patients needed intensive care management (27/79), and surgical intervention (35/79), and the fatality rate was 5.1% (4/79). Genomic sequencing (n = 55) revealed the dominant genetic lineage by the M1_UK_-lineage (26/55). In a Belgian multicenter case series, a total of 86 patients (56 adults; 30 children) with *S. pyogenes* infections were enrolled [17]. There was an incidence of severe pneumonia (45% of adults; 77% of children) with empyema: two-thirds of pneumonia patients had viral co-infection, with influenza (13 adults; five children). Other disease manifestations contained NSTI (23% of adults) and other severe skin/soft tissue infections (16% of adults; 13% of children). Mortality was 21% among adults and 3% among children. Genomic analysis (n = 55) showed the predominance of the *emm1 *clone (73%), mostly by the M1_UK_ lineage (83% of *emm1*). Therefore, clinicians should pay special attention to the onset of pneumonia as well as skin/soft tissue infections during viral infection season(s). 

**Table 1:** A literature review including four main case reports of M1UK-associated infections among adults STSS: streptococcal toxic shock syndrome; COVID-19: coronavirus disease 2019

Publication year (reference no.)	Country	Sex/age (years)	Comorbidities	Isolation source(s)	Clinical diagnosis	Initial antimicrobial(s) [dose]	Antimicrobial susceptibility	Surgical intervention(s)	Other intervention(s)	Outcome
2024 [14]	Greece	Female/40	Healthy	Pleural fluid & sputum	Pneumonia, empyema, & STSS	Piperacillin/tazobactam & linezolid	Resistant to erythromycin & clindamycin	Chest tube thoracostomy	Invasive positive pressure ventilation, continuous renal replacement therapy, continuous venovenous hemodiafiltration, CytoSorb therapy, & intra-aortic balloon pumping	Cured
2024 [10]	Japan	Male/71	COVID-19 & nasopharyngeal cancer	Sputum	Pneumonia following COVID-19	Ampicillin/sulbactam (12 g/day) & levofloxacin (0.5 g/day)	Susceptible to all validated antimicrobials	None	Invasive positive pressure ventilation & extracorporeal membrane oxygenation	Cured
2024 [10]	Japan	Male/91	COVID-19 & thyroid cancer	Pericardial fluid	Pericarditis & mediastinitis following COVID-19	Ampicillin/sulbactam (12 g/day)	Susceptible to all validated antimicrobials	Pericardiocentesis/percutaneous pericardial drainage	None	Cured
This study	Japan	Male/34	Atopic dermatitis	Deep effusion & sputum	Necrotizing soft tissue infection & bronchitis	Meropenem (3 g/day) & clindamycin (2.7 g/day)	Susceptible to all validated antimicrobials	Debridement	Negative pressure wound therapy with instillation and dwelling, micrografting, & split-thickness skin grafting	Cured

The National Institution of Infectious Diseases has reported risk assessment for STSS in Japan in 2024, and a nationwide increase in M1_UK_-lineage among the STSS subjects [18]. In autumn 2024, our medical institute is examining and treating patients with *S. pyogenes* infections. The Infectious Agents Surveillance Report from Saitama prefecture, where the medical institute is located, has documented 17 cases of M1_UK_-associated STSS between June 2023 and May 2024 [19]. Recently, Golden et al. published an invasive group A Streptococcus hypervirulent M1_UK_ clone, Canada, 2018-2023 [20]. The percentage of toxigenic and hypervirulent M1_UK_-lineage strains increased significantly (from 22.1% in 2018 to 60.2% in 2023). The genomic investigation identified the geographically and temporally associated clusters and genes associated with virulent bacteriophage acquisition. Therefore, we further need to conduct the cohort study to assess the incidences of M1_UK_, M1_intermediate_, and M1_global_ among *emm*1/M1 strains.

## Conclusions

Early suspicion of NSTI is crucial for timely diagnosis and management. Prompt exploratory incision is strongly recommended for both the diagnosis and treatment of this condition. Effective treatment of NSTI requires debridement of necrotic tissue, followed by reconstruction of the resulting tissue defects. Based on the clinical experience of this case, micrografting using the Rigenera® system and subsequent STG, which is combined with NPWTi-d to promote the patient’s local cure, can be a beneficial approach.

We identified an M1_UK_-lineage *S. pyogenes* variant using the fascia effusions in this case, although two sets of blood cultures upon admission revealed no bacterial growth. Therefore, clinicians should perform cultures using sterile specimens (i.e., deep effusions or tissues) from infection foci through exploratory incision or debridement, in addition to two sets of blood cultures, when examining and treating patients with or without underlying medical conditions who are suspected to develop NSTIs/non-NSTIs.
